# Analysis of the Genetic Diversity Associated With the Drug Resistance and Pathogenicity of Influenza A Virus Isolated in Bangladesh From 2002 to 2019

**DOI:** 10.3389/fmicb.2021.735305

**Published:** 2021-09-17

**Authors:** Md. Golzar Hossain, Sharmin Akter, Priya Dhole, Sukumar Saha, Taheruzzaman Kazi, Abir Majbauddin, Md. Sayeedul Islam

**Affiliations:** ^1^Department of Microbiology and Hygiene, Bangladesh Agricultural University, Mymensingh, Bangladesh; ^2^Department of Physiology, Bangladesh Agricultural University, Mymensingh, Bangladesh; ^3^Department of Biology, The Pennsylvania State University, Pennsylvania, PA, United States; ^4^Department of Regenerative Dermatology, Graduate School of Medicine, Osaka University, Osaka, Japan; ^5^Department of Biological Sciences, Graduate School of Science, Osaka University, Osaka, Japan

**Keywords:** influenza A virus, subtypes, host, mutations, drug resistance, pathogenicity, Bangladesh

## Abstract

The subtype prevalence, drug resistance- and pathogenicity-associated mutations, and the distribution of the influenza A virus (IAV) isolates identified in Bangladesh from 2002 to 2019 were analyzed using bioinformatic tools. A total of 30 IAV subtypes have been identified in humans (4), avian species (29), and environment (5) in Bangladesh. The predominant subtypes in human and avian species are H1N1/H3N2 and H5N1/H9N2, respectively. However, the subtypes H5N1/H9N2 infecting humans and H3N2/H1N1 infecting avian species have also been identified. Among the avian species, the maximum number of subtypes (27) have been identified in ducks. A 3.56% of the isolates showed neuraminidase inhibitor (NAI) resistance with a prevalence of 8.50, 1.33, and 2.67% in avian species, humans, and the environment, respectively, the following mutations were detected: V116A, I117V, D198N, I223R, S247N, H275Y, and N295S. Prevalence of adamantane-resistant IAVs was 100, 50, and 30.54% in humans, the environment, and avian species, respectively, the subtypes H3N2, H1N1, H9N2, and H5N2 were highly prevalent, with the subtype H5N1 showing a comparatively lower prevalence. Important PB2 mutations such D9N, K526R, A588V, A588I, G590S, Q591R, E627K, K702R, and S714R were identified. A wide range of IAV subtypes have been identified in Bangladesh with a diversified genetic variation in the NA, M2, and PB2 proteins providing drug resistance and enhanced pathogenicity. This study provides a detailed analysis of the subtypes, and the host range of the IAV isolates and the genetic variations related to their proteins, which may aid in the prevention, treatment, and control of IAV infections in Bangladesh, and would serve as a basis for future investigations.

## Introduction

Influenza caused by influenza viruses (types A, B, C, and D) is a contagious respiratory infection distributed worldwide. Influenza has a wide host range depending on the strains of the influenza A virus (IAV). Based on the circulating strains of the influenza virus, a myriad range of hosts have been described. Many avian and mammalian species such as chicken, duck, quail, and crow, and human, swine, horse, and cat, respectively, might be infected with IAV (Taubenberger and Kash, 2010; Hussain et al., 2017). Every year, millions of people are infected with influenza worldwide, with increasing economic losses due to diagnosis, treatment, and vaccine development costs (Molinari et al., 2007; Mao et al., 2012; Palekar et al., 2019). Likewise, some strains of the IAV with a high morbidity and mortality rate severely impact the poultry industries (Thomas and Noppenberger, 2007; Yoo et al., 2018).

IAV is an enveloped virus of around 100-nm diameter. It belongs to the *Alphainfluenzavirus* genus and *Orthomyxoviridae* family. It is a segmented RNA virus, which is negative-sense, single-stranded, and contains eight RNA segments (Eisfeld et al., 2015). The entire genome is around 13,588 bases encoding at least 10 viral proteins (Eisfeld et al., 2015). The major proteins encoded by the viral genomes are hemagglutinin (HA), neuraminidase (NA), nucleoprotein (NP), matrix proteins (M1 and M2), polymerase proteins (PB1, PB2, and PA), and non-structural proteins (NS1 and NEP) (McCauley and Mahy, 1983).

The HA and NA proteins are present on the outermost surface of the virion and play crucial roles in the attachment of the virus to the host cells to initiate the infection cycle, leading to the subsequent release of the viral progeny (Suzuki, 2005; Cohen et al., 2013). The currently available anti-influenza drugs primarily target the NA protein (Wilson and von Itzstein, 2003). The IAV can be classified into different subtypes based on the antigenic properties of the HA and NA proteins. To date, 16 HA and 9 NA subtypes have been reported based on their amino acid variations (Lynch and Walsh, 2007; Proença-Módena et al., 2007). The HA protein determines the host specificity of IAV. The HA proteins of the avian and human influenza viruses bind with two to three sialic acid and two to three sialic acid receptors, respectively, whereas the swine influenza binds both (Trebbien et al., 2011; Byrd-Leotis et al., 2017). However, many of IAV subtypes, H5N1, H7N9, and H9N2, can infect both birds and humans (Mostafa et al., 2018). On the other hand, the M2 protein activated during the IAV entry into the host cells releases the ribonucleoprotein into the cytoplasm to initiate the viral replication (Jalily et al., 2020). The drug adamantane blocks the viral replication during the uncoating step by inhibiting the IAV M2 ion transport (Takeda et al., 2002; Jalily et al., 2020). However, mutations in the NA and M2 proteins might affect the drug susceptibility, and resistant IAV strains have been reported (Hussain et al., 2017). Drug-resistant and mutated highly pathogenic IAVs are distributed worldwide (Wen et al., 2018; Moasser et al., 2019).

Surveillance, vaccines, and antiviral drugs are essential to control and eradicate IAV from a region (Hussain et al., 2017). The rapid spread of drug-resistant IAVs interrupts the management and prevention strategies (Ormond et al., 2017). Hence, researchers have been investigating and designing various next-generation antivirals that are effective against drug-resistant IAVs (Shin and Seong, 2019; Musharrafieh et al., 2020; Toots and Plemper, 2020; Yin et al., 2021). In addition, current influenza vaccines provide only partial protection and may fail to protect against the mutated and novel variants of IAVs (Vanderlinden and Naesens, 2014; Paules and Fauci, 2019). Therefore, the surveillance and analysis of the IAV mutations associated with drug resistance are important for the prevention and treatment of IAV infection.

Among the three subunits of IAV polymerases (PB1, PB2, and PA), PB2 is an important virulence determinant (Graef et al., 2010). PB2 initiates the genome replication of IAV by modulating the ribonucleoprotein complex and regulating the host immune system and antiviral signaling pathways (Carr et al., 2006; Graef et al., 2010). Many clinical and experimental studies have shown that mutations in PB2 affect the activity of this polymerase and the pathogenicity of IAV (Tian et al., 2012; de Jong et al., 2013; Song et al., 2014; Wen et al., 2018). PB2 mutations are also responsible for the interspecies transmission of IAV (Yamada et al., 2010; Wen et al., 2018; Wang et al., 2019). Therefore, the mutational analysis of PB2 in terms of the pathogenicity and interspecies transmission of IAV will be very helpful in preventing and controlling IAV infections.

Human and avian IAV infections were reported in Bangladesh in 2002 and 2006, respectively (Chi et al., 2005; Rimi et al., 2019). On the other hand, highly pathogenic avian influenza (HPAI) H5N1 was reported in poultry in 2007 for the first time and posed a serious threat to public health (Rimi et al., 2019). Moreover, HPAI H5N1 and low pathogenic avian influenza (LPAI) H9N2 caused significant damage to the poultry industry in Bangladesh (Turner et al., 2017; Kim, 2018; Parvin et al., 2018). In addition, the genetic reassortment of HPAI and the introduction of the new clades of IAV are now very common in Bangladesh (Parvin et al., 2014; Marinova-Petkova et al., 2016; Nooruzzaman et al., 2019). However, to our knowledge, the overall prevalence of the different IAV subtypes and their host range in Bangladesh have not yet been reported. The analysis of the genetic mutations associated with drug resistance and pathogenicity is necessary for the development of vaccines and treatment strategies to eradicate IAV from Bangladesh. Therefore, this study analyzed the overall prevalence of the IAV subtypes in Bangladesh from 2002 to 2019 along with the determination of their specific host distribution. Moreover, mutational analyses were performed to determine the prevalence of the drug resistance-associated mutations in the NA and M2 proteins and the pathogenicity-associated mutations in the PB2 protein of IAV.

## Materials and Methods

### Collection and Processing of Influenza A Virus Sequences

The genome sequences of the IAV isolates from Bangladesh were retrieved from GISAID.^1^ The data about IAV isolates deposited between 2002 and 2019 in GISAID was acquired. The sequences were either full or partial. The IAV isolates from Bangladesh were cross-checked with the NCBI influenza virus database (Bao et al., 2007) and Influenza Research Database.^2^ The retrieved sequences were downloaded, processed, and analyzed using several bioinformatic tools, such as CLC Sequence viewer,^3^ NCBI influenza virus database (Bao et al., 2007), and Influenza Research Database (see text footnote 2). The CLC Sequence viewer is a basic bioinformatics tool used to view, create, and edit alignments, and analyze the genomic sequences. The Influenza Virus Sequence Annotation Tool of the NCBI influenza virus database is an online application that can predict influenza protein sequences coded by the nucleotide sequences with specific drug resistance- and virulence-associated mutations. The Influenza Research Database contains surveillance data of the non-human and avian species and the clinical data of humans with the phenotypic, genomic, and proteomic data of the isolated virus strain. This database is used to analyze and visualize various features such as alignment, point mutations in specific proteins, and drug resistance (see text footnote 2).

### Analysis of the Prevalence of the Influenza A Virus Subtypes

A total of 2,005, either full or partial genome sequences of IAV isolates, were used to determine subtype prevalence. According to the GenBank and NCBI Influenza Virus Database, a few sequences were designated as mixed by the sequence submitter. On the other hand, the subtype of some sequences could not be determined by the sequence submitter. Therefore, these mixed or undetermined subtypes were excluded from the analysis. The sequences were screened either using the NCBI influenza virus database or Microsoft Excel for the specific subtypes and their host distribution, and the prevalence was analyzed accordingly.

### Analysis of Drug Resistance-Associated Mutations in Neuraminidase and M2 Proteins

A total of 1,828 amino acid sequences of the viral NA protein of the IAV isolates were analyzed for mutations associated with neuraminidase inhibitor (NAI) resistance. Among these, 1,200, 553, and 75 sequences were from human-, avian-, and environment-derived IAVs, respectively. The mutations were determined using the “Antiviral Resistance Risk Assessment” tool of the Influenza Research Database (Zhang et al., 2016). Based on the previously submitted data, this tool determines the amino acid mutations in the sequences associated with the alteration of susceptibility to antiviral drugs. A total of 1,761 amino acid sequences of M2 protein were retrieved (human: 1,220, avian: 465, and environment: 76), and adamantane resistance-associated mutations were analyzed using the “Identify Point Mutations in Proteins” tool of the Influenza Research Database (Bao et al., 2007; Dong et al., 2015). This tool had three parameters set as default: type of protein to scan for the specified mutation (s), specific mutation coordinate, and specific subtype (s). Within these parameters, the subtype, protein, and mutation name with their amino acid positions were changed every time based on the purpose of the analysis.

### Analysis of the PB2 Amino Acid Mutations Associated With Influenza A Virus Virulence

In total, 1,568 amino acid sequences of the PB2 protein were analyzed for the mutations associated with IAV virulence using the “Identify Point Mutations in Proteins” tool of the Influenza Research Database (Bao et al., 2007; Kim et al., 2010; Wen et al., 2018). Of these, 1,050, 456, and 62 sequences were from human-, avian-, and environment-derived IAVs.

## Results

### Prevalence of Influenza A Virus Subtypes in the Human, Environmental, and Avian Species

**FIGURE 1:**
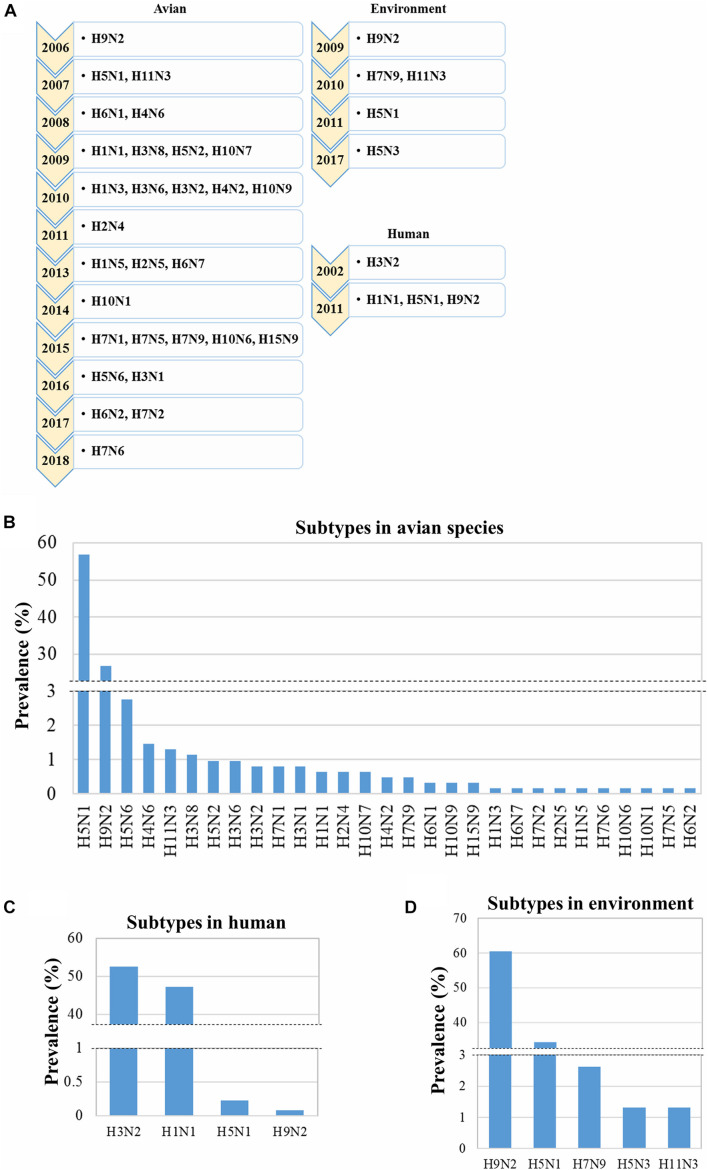
Emergence and prevalence of influenza A virus (IAV) subtypes in humans, environment, and avian species. Data regarding 2,005 IAV isolates (human: 1,311, avian: 618, and environment: 76) collected in Bangladesh from 2002 to 2019 were retrieved from GISAID, and the prevalence of the different subtypes was determined. **(A)** Year of first reporting of the emergence of subtypes from 2002 to 2019. Prevalence of IAV subtypes in the avian hosts **(B)**, humans **(C)**, and environment **(D)**.

The human IAVs were first reported in Bangladesh in 2002, whereas avian influenza was reported in 2006, according to the GenBank information (Chi et al., 2005; Bao et al., 2007; Parvin et al., 2014). Furthermore, a highly pathogenic IAV (H5N1) was reported in 2007 (Rimi et al., 2019). However, during the last two decades, many IAV subtypes have been reported sporadically in Bangladesh. Therefore, the overall prevalence of the identified subtypes in Bangladesh, from 2002 to 2019 from a total of 2,005 IAV isolates, was analyzed (human: 1,311, avian: 618, and environment: 76). To date, 30 subtypes of IAV have been identified in Bangladesh among which only 4 infect humans, 5 were from non-human and non-avian environmental sources, and 29 subtypes infect avian species (Figure 1A). Among the avian IAV subtypes, the subtype H5N1 is highly prevalent (56.80%) followed by H9N2 (26.70%) (Figure 1B). The prevalence of the subtypes H3N2 (52.48%) and H1N1 (47.22%) in humans was higher though sporadic infections were found with H5N1 (0.23%) and H9N2 (0.08%) (Figure 1C). However, in the environment, the prevalence of H9N2 was higher (60.53%) compared with that of H5N1 (34.21%), H7N9 (2.63%), H5N3 (1.32%), and H11N3 (1.32%) (Figure 1D). In summary, among the 30 IAV subtypes circulating in Bangladesh, H5N1 and H9N2 predominantly infect avian hosts or are primarily isolated from the environment, whereas H3N2 and H1N1 infect humans. The subtypes H5N1 and H9N2 were also sporadically identified in humans though they are primarily found in the environment and avian species.

### Distribution of the Influenza A Virus Subtypes in Various Species of Birds

**FIGURE 2:**
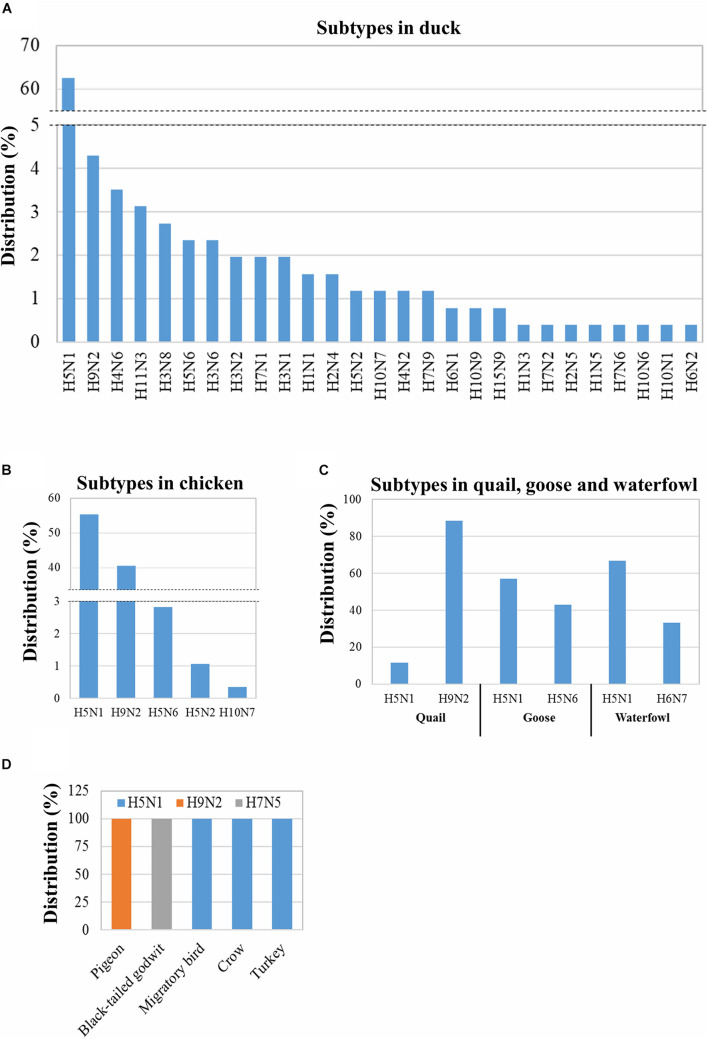
Distribution of the IAV subtypes in various avian species in Bangladesh. Distribution was analyzed using 618 IAV isolates from 2006 to 2019 retrieved from GISAID. Distribution of 256 IAV duck isolates into subtypes **(A)**. Distribution of 284 chicken, 43 quail, 7 goose, and 3 waterfowl IAV isolates among subtypes **(B,C)**. Distribution of mentioned avian IAV isolates in different subtypes **(D)**.

As 29 out of the reported 30 subtypes of IAV (618 isolates) have been found in avian species, the distribution of the specific subtypes in different avian hosts was analyzed. These 29 subtypes can infect 10 different avian species (Figure 2). The maximum number of subtypes (27) were isolated from ducks followed by chickens (5) and two each were found in quails, geese, and waterfowls; the remaining five avian species were each found to host only one subtype (Figure 2). Moreover, H5N1 was found to be distributed among the highest number of avian species (eight), followed by H9N2 (four species), H5N6 (three species), and H5N2 and H10N7 (two species each) (Figure 2). Next, the prevalence of the different subtypes in the specific avian hosts was analyzed. From the total of 27 IAV subtypes identified in ducks, 62.5% were H5N1 (Figure 2A). In addition, among the five subtypes circulating in chicken, H5N1 (55%) and H9N2 (40.49%) were found to be highly prevalent (Figure 2B). However, at least two subtypes were identified in quails, geese, and waterfowls (Figure 2C). The other five avian species showed the prevalence of only one subtype of IAV (Figure 2D). In conclusion, though 29 IAV subtypes were identified in the avian hosts in Bangladesh, the subtypes H5N1 and H9N2 were found to be the most predominant. A large number of subtypes (27) were determined to be circulating in ducks.

### Analysis of Neuraminidase Inhibitor Resistance-Associated Mutations

**FIGURE 3:**
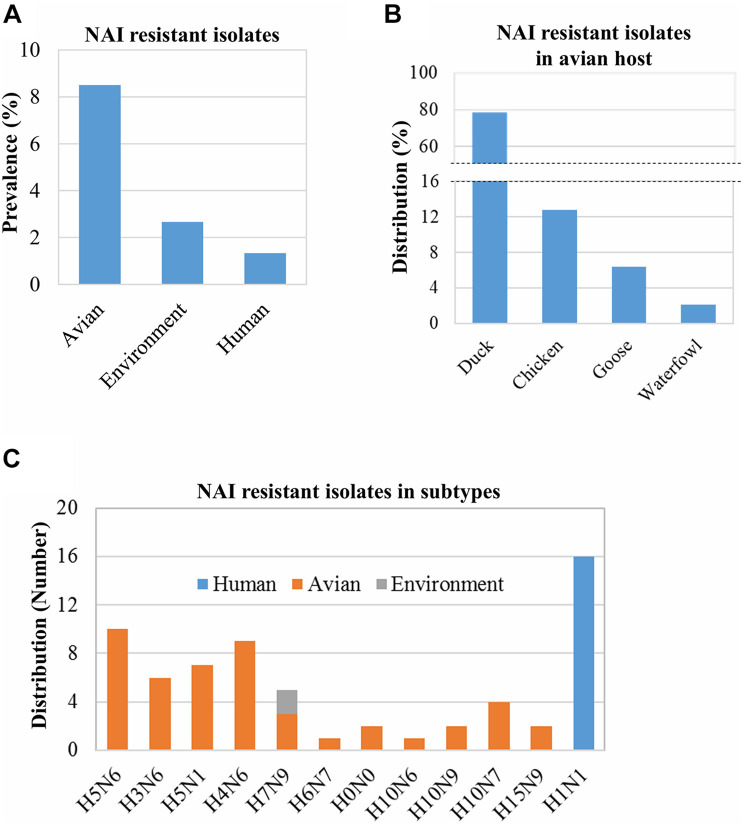
Prevalence of NAI resistance-associated mutations and their distribution in hosts and virus subtypes. The prevalence of NAI resistance-associated mutations among 1,828 isolates/sequences (human: 1,200, avian: 553, and environment: 75) was analyzed using the “Antiviral Resistance Risk Assessment” tool of the Influenza Research Database **(A)**. The distribution of 47 NAI-resistant isolates among avian species was analyzed **(B)**. The distribution of 65 isolates found resistant to NAI in humans, avian hosts, and the environment **(C)**. H0N0 means mixed isolates.

Some IAV isolates show resistance against NAIs due to mutations in their neuraminidase (NA) proteins (Abed et al., 2006; Zürcher et al., 2006; Orozovic et al., 2011; WHO, 2018; Moasser et al., 2019). Based on the analysis of 1,828 isolates/sequences of IAV NA proteins, we found 65 IAV isolates showing resistance to oseltamivir and two showing resistance to oseltamivir and zanamivir. The overall prevalence of NAI-resistant IAV isolates was 3.56% (65/1,828). The prevalence in humans, birds, and the environment was 1.33% (16/1220), 8.50% (47/553), and 2.67% (2/75), respectively (Figure 3A). The distribution of the NAI-resistant human and environmental isolates was limited to H1N1 and H7N9. The avian IAV isolates resistant to NAI were distributed among a wide range of subtypes (Figure 3C). Among 47 NAI-resistant avian IAV isolates, 78.72% (37/47) were found in ducks, 12.76% in chickens, 6.38% in geese, and 2.12% in waterfowls (Figure 3B). The corresponding mutations were V116A, I117V, D198N, D199M, I223R, S247N, H275Y, and N295S, which were previously shown to occur naturally in the infected hosts (Table 1).

**TABLE 1:** Mutations associated with the drug resistance and pathogenicity of the influenza A virus (IAV) isolates found in this study.

Proteins	Mutations	Properties
NA	V116A	Resistance to oseltamivir or zanamivir
	I117V	
	D198N	
	D199N	
	I223R	
	S247N	
	H275Y	
	N295S	
M2	L26F	Resistance to adamantanes
	V27A	
	S31N	
PB2	D9N	Increased pathogenicity
	K526R	
	A588I/V	
	G590S/Q591R	
	E627K	
	K702R	
	S714R	

### Analysis of the Mutations in the M2 Protein Associated With Adamantane Resistance

**FIGURE 4:**
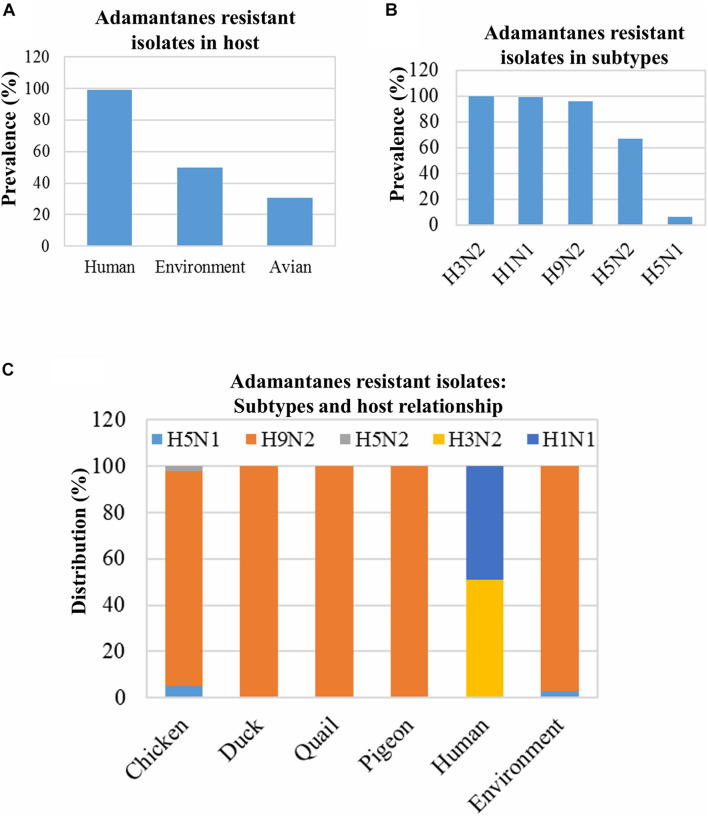
Prevalence of adamantane resistance-associated mutations and their distribution in host and virus subtypes. Isolates, 1,761 (human: 1,220, avian: 465, and environment: 76), were screened for M2 protein mutations associated with adamantane resistance, and prevalence was analyzed **(A)**. The prevalence of adamantane-resistant mutations among 1,489 isolates of the abovementioned subtypes was analyzed **(B)**. The distribution of all 1389 isolates showed adamantanes resistant mutations among the different hosts **(C)**.

Several mutations, such as L26F, V27A, A30T, A30V, S31N, and G34E in the M2 protein, have been reported to be associated with the adamantane resistance of IAVs isolated from infected hosts (Bao et al., 2007; Dong et al., 2015). The prevalence of adamantane-resistant isolates was 78.88% (1389/1761); these isolates were limited to only five subtypes: H9N2, H5N1, H3N2, H1N1, and H5N2 (Figure 4B). Almost 100% (1,209/1,220) of the human IAV isolates from Bangladesh were found to be resistant to adamantanes, followed by the environment-derived IAVs (50%; 38/76), and the avian host-derived IAVs (30.54%; 142/465) (Figure 4A). Moreover, almost 100% of the H3N2, H1N1, and H9N2 subtypes were adamantane-resistant, whereas the least number of the H5N1 isolates (6.67%) showed resistance to adamantanes (Figure 4B). Besides, in the case of avian isolates, the adamantane-resistant IAVs were found only in chickens, ducks, quails, and pigeons (Figure 4C).

### Mutation in the PB2 Protein Associated With Virulence of Influenza A Viruses

**FIGURE 5:**
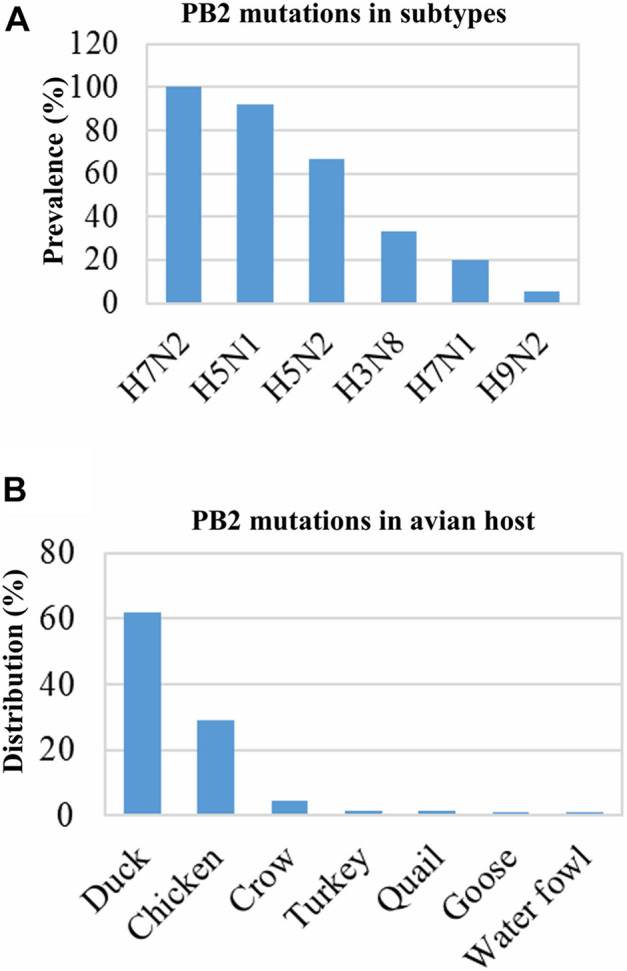
Prevalence of PB2 protein mutations associated with increased pathogenicity and distribution in host and subtypes. Data of 1,568 isolates (human: 1,050, avian: 456, and environment: 62) were retrieved and analyzed. In avian hosts, 221 isolates showed PB2 mutations associated with increased pathogenicity, which were from 376 isolates of the abovementioned subtypes **(A)**. Distribution of the 221 isolates among different avian species **(B)**.

Mutations in the protein PB2 increase the viral polymerase activity and pathogenicity of the IAV (Tian et al., 2012; de Jong et al., 2013; Song et al., 2014; Wen et al., 2018). Our analysis showed that almost 100% of the human IAV isolates contained PB2 mutations. These mutations are required for adaptation in mammalian hosts, and many of them might be associated with the increased pathogenicity. Nearly all of these mutations belonged to H1N1 (46.19%) and H3N2 (53.52%). The prevalence of mutated IAV isolates with increased pathogenicity was 48.46% (221/456) and 30.65% (19/62) in the avian host and environment, respectively. The mutations were found in the H5N1 (208/226), H9N2 (7/135), H5N2 (2/3), H3N8 (2/6), H7N1 (1/7), and H7N2 (1/1) isolates. Most of the PB2 mutations of avian IAVs were found in H5N1 (208/226) and were primarily distributed in ducks (62%) and chickens (28.96%) (Figures 5A,B). All environmental isolates of H5N1 contain mutated PB2. Several mutations were identified in the sequences of the IAV isolates, namely, D9N, K526R, A588I/V, G590S/Q591R, E627K, K702R, and S714R. These mutations were previously reported to be associated with increased pathogenicity (Table 1; Tian et al., 2012; de Jong et al., 2013; Song et al., 2014; Wen et al., 2018). However, many isolates contained multiple mutations in PB2, especially isolates of human IAVs.

## Discussion

A total of 16 HA and 9 NA subtypes have been identified worldwide, and many of these have been reported as zoonotic (Proença-Módena et al., 2007; Mostafa et al., 2018). Several subtypes (H1N1, H2N2, and H3N2) of IAV are pandemic in humans, but genetic reassortment of other zoonotic subtypes may increase the risk of infection in humans (Taubenberger and Kash, 2010; Chastagner et al., 2019). In the last few decades, mutations in the IAV genes have made the selection of appropriate antiviral drugs tremendously challenging. Therefore, proper IAV subtyping is essential for selecting the appropriate antiviral drug to cure the specific IAV infection and to identify new therapeutic targets, as several subtypes are resistant to the currently available antiviral drugs (Kaul et al., 2010; Parida et al., 2016). Moreover, IAV vaccine efficacy may vary depending on the virus strain such as against a specific subtype, H3N2 (Belongia et al., 2016). IAV infects a wide range of hosts, and interspecies transmission is possible (Joseph et al., 2017). In this study, 30 subtypes of IAV were identified in Bangladesh from 2002 to 2019. Of these, 29 were found in avian species with a predominance of H5N1 and H9N2. Four human IAV isolates were reported, with 99.7% belonging to H1N1 and H3N2 (Komadina et al., 2014). However, the interspecies transmission and genetic reassortment of IAVs occurred in Bangladesh and correlated with the global cases (Zhou et al., 2011; Lai et al., 2016; Briand et al., 2018). Interestingly both H3N2 and H1N1 were identified only in ducks, which were found to be infected with a total of 27 subtypes of IAVs in Bangladesh (Wilcox et al., 2011). Ducks might be reservoirs of various IAV subtypes, and coinfection with more than one subtype may be very common, and may facilitate the emergence of a more pathogenic new strain of IAV following genetic reassortment (Hinshaw et al., 1980; Sharp et al., 1997; Wilcox et al., 2011; Deng et al., 2013). Moreover, the emergence of H3N2 from ducks after genetic reassortment with other subtypes is alarming as novel virus strains infecting humans or other mammals may emerge (Zhou et al., 2011; El-Shesheny et al., 2018).

IAV-infected patients can be treated with two groups of anti-IAV drugs; adamantanes that block the viral M2 ion channel activity and NAIs, which inhibit the enzymatic activities of the viral NA protein (Hussain et al., 2017; Jalily et al., 2020; Musharrafieh et al., 2020). However, mutated adamantane-resistant IAV strains have been emerging since 1980 due to mutations in the M2 protein, and their prevalence is increasing (Heider et al., 1981; Dong et al., 2015). The IAV subtypes circulating in humans worldwide, especially the H1N1, and H3N2, show significant resistance to amantadine (Hussain et al., 2017). Few reports showed that the prevalence of adamantane-resistant IAVs exceeds 90% in Asian countries (Nelson et al., 2009; Dong et al., 2015). Accordingly, more than 99% of the human isolates in this analysis showed resistance to adamantanes, and they belong to the H1N1 and H3N2 subtypes. Moreover, more than 90% of H9N2 isolates from humans, avian hosts, and the environment also showed adamantane resistance. In addition, a small percentage of avian and environmental H5N1 isolates were also found to be resistant to adamantanes, consistent with previous findings (Cheung et al., 2006; Govorkova et al., 2013).

Though several NAIs are available in the market, oseltamivir and zanamivir have been used worldwide (Principi et al., 2019). However, mutations in the NA protein affecting the sensitivity of these drugs and IAV resistance have also emerged (Abed et al., 2006; Zürcher et al., 2006; Hussain et al., 2017; WHO, 2018; Moasser et al., 2019). During the study period of 2002 to 2019, 3.56% of the IAV isolates have been found to be resistant to oseltamivir. Interestingly the resistance rate is higher in avian species than in humans. All of the oseltamivir-resistant IAVs were of the H1N1 subtype, which is supported by the findings of a previous global analysis (Hussain et al., 2017). However, a wide range of IAV subtypes with NAI resistance were primarily found in ducks (Earhart et al., 2009; Järhult, 2012; Achenbach and Bowen, 2013).

Many mutations in the PB2 protein identified in human IAV isolates are present in avian species confirming the possibility of interspecies transmission (Yamada et al., 2010; Wen et al., 2018; Wang et al., 2019). PB2 mutations E627K and D701N are found in H5N1 among human isolates, whereas avian type PB2 exhibits the mutations 526K, 627E, and 701D (Chen et al., 2005; Mehle and Doudna, 2008). The PB2 K526R mutation is found in H3N2 among mammalian isolates, whereas the H1N1 subtype shows the mutation 590S/591R for replication in humans (Mehle and Doudna, 2009; Song et al., 2014). PB2 K702R is also supposed to be a host marker as it is found in human H5N1 in Indonesia and has not been reported in avian species (Finkelstein et al., 2007). The mutational analysis of human IAV viral PB2 polymerase from 2002 to 2019 showed that all H1N1 subtypes contained G590S/Q591R mutations, whereas almost all of the H3N2 subtypes contained K526R, E627K, and K702R simultaneously. Interestingly, more than 90% of the avian and 100% of the environmental H5N1 PB2 also contained the K526R, E627K, or K702R mutations, and these mutations were mostly distributed in ducks and chickens. This might be due to interspecies transmission and natural selection (Wang et al., 2019).

In summary, a total of 30 subtypes of the IAV were identified in Bangladesh. Among them, H5N1 and H9N2, are predominant in the avian host and environment, whereas H3N2 and H1N1 are prevalent in humans. The subtypes H1N1, H5N1, and H9N2 have been isolated from humans and avian species. A wide range of subtypes (27) has been found in ducks. All human IAV isolates are resistant to adamantanes. Though the prevalence is low, NAI-resistant IAVs have been circulating in Bangladesh. A high number of avian isolates contained PB2 mutations associated with increased pathogenicity in the human IAV.

## Author Contributions

MGH designed the study, extracted the sequences, data, and information from the online databases, analyzed the data, and wrote the manuscript. MGH, SA, PD, SS, TK, AM, and MSI edited, revised, and approved the final version of the manuscript. All authors contributed to the article and approved the submitted version.

## Conflict of Interest

The authors declare that the research was conducted in the absence of any commercial or financial relationships that could be construed as a potential conflict of interest.

## Publisher’s Note

All claims expressed in this article are solely those of the authors and do not necessarily represent those of their affiliated organizations, or those of the publisher, the editors and the reviewers. Any product that may be evaluated in this article, or claim that may be made by its manufacturer, is not guaranteed or endorsed by the publisher.

## Footnotes

1
https://www.gisaid.org/

2
https://www.fludb.org/

3
http://www.clcbio.com

## References

[B1] Abed Y. Baz M. Boivin G. (2006). Impact of neuraminidase mutations conferring influenza resistance to neuraminidase inhibitors in the N1 and N2 genetic backgrounds. *Antivir. Ther.* 11 971–976. 17302366

[B2] Achenbach J. E. Bowen R. A. (2013). Effect of oseltamivir carboxylate consumption on emergence of drug-resistant H5N2 avian influenza virus in Mallard ducks. *Antimicrob. Agents Chemother.* 57 2171–2181. 10.1128/AAC.02126-12 23459475 PMC3632944

[B3] Bao Y. Bolotov P. Dernovoy D. Kiryutin B. Tatusova T. (2007). FLAN: a web server for influenza virus genome annotation. *Nucleic Acids Res.* 35 W280–W284. 10.1093/nar/gkm354 17545199 PMC1933127

[B4] Belongia E. A. Simpson M. D. King J. P. Sundaram M. E. Kelley N. S. Osterholm M. T. (2016). Variable influenza vaccine effectiveness by subtype: a systematic review and meta-analysis of test-negative design studies. *Lancet Infect. Dis.* 16 942–951. 10.1016/S1473-3099(16)00129-8 27061888

[B5] Briand F.-X. Niqueux E. Schmitz A. Beven V. Lucas P. Allée C. (2018). Identification of a divergent avian influenza H3N2 virus from domestic ducks in France. *Microbiol. Resour. Announc.* 7 e00943–18. 10.1128/MRA.00943-18 30574576 PMC6298543

[B6] Byrd-Leotis L. Cummings R. D. Steinhauer D. A. (2017). The Interplay between the host receptor and influenza virus hemagglutinin and neuraminidase. *Int. J. Mol. Sci.* 18:1541. 10.3390/ijms18071541 28714909 PMC5536029

[B7] Carr S. M. Carnero E. García-Sastre A. Brownlee G. G. Fodor E. (2006). Characterization of a mitochondrial-targeting signal in the PB2 protein of influenza viruses. *Virology* 344 492–508. 10.1016/j.virol.2005.08.041 16242167

[B8] Chastagner A. Bonin E. Fablet C. Quéguiner S. Hirchaud E. Lucas P. (2019). Virus persistence in pig herds led to successive reassortment events between swine and human influenza A viruses, resulting in the emergence of a novel triple-reassortant swine influenza virus. *Vet. Res.* 50:77. 10.1186/s13567-019-0699-y 31590684 PMC6781375

[B9] Chen H. Smith G. J. D. Zhang S. Y. Qin K. Wang J. Li K. S. (2005). H5N1 virus outbreak in migratory waterfowl. *Nature* 436 191–192. 10.1038/nature03974 16007072

[B10] Cheung C.-L. Rayner J. M. Smith G. J. D. Wang P. Naipospos T. S. P. Zhang J. (2006). Distribution of amantadine-resistant H5N1 avian influenza variants in asia. *J. Infect. Dis.* 193 1626–1629. 10.1086/504723 16703504

[B11] Chi X. S. Bolar T. V. Zhao P. Tam J. S. Rappaport R. Cheng S.-M. (2005). Molecular evolution of human influenza A/H3N2 virus in Asia and Europe from 2001 to 2003. *J. Clin. Microbiol.* 43 6130–6132. 10.1128/JCM.43.12.6130-6132.2005 16333111 PMC1317174

[B12] Cohen M. Zhang X. Q. Senaati H. P. Chen H. W. Varki N. M. Schooley R. T. (2013). Influenza A penetrates host mucus by cleaving sialic acids with neuraminidase. *Virol. J.* 10:321. 10.1186/1743-422X-10-321 24261589 PMC3842836

[B13] de Jong R. M. C. Stockhofe-Zurwieden N. Verheij E. S. De Boer-Luijtze E. A. Ruiter S. J. M. De Leeuw O. S. (2013). Rapid emergence of a virulent PB2 E627K variant during adaptation of highly pathogenic avian influenza H7N7 virus to mice. *Virol. J.* 10:276. 10.1186/1743-422X-10-276 24007444 PMC3766704

[B14] Deng G. Tan D. Shi J. Cui P. Jiang Y. Liu L. (2013). Complex reassortment of multiple subtypes of avian influenza viruses in domestic ducks at the Dongting lake region of China. *J. Virol.* 87:9452. 10.1128/JVI.00776-13 23804642 PMC3754128

[B15] Dong G. Peng C. Luo J. Wang C. Han L. Wu B. (2015). Adamantane-resistant influenza a viruses in the world (1902-2013): frequency and distribution of M2 gene mutations. *PLoS One* 10:e0119115. 10.1371/journal.pone.0119115 25768797 PMC4358984

[B16] Earhart K. C. Elsayed N. M. Saad M. D. Gubareva L. V. Nayel A. Deyde V. M. (2009). Oseltamivir resistance mutation N294S in human influenza A(H5N1) virus in Egypt. *J. Infect. Public Health* 2 74–80. 10.1016/j.jiph.2009.04.004 20701864

[B17] Eisfeld A. J. Neumann G. Kawaoka Y. (2015). At the centre: influenza A virus ribonucleoproteins. *Nat. Rev. Microbiol.* 13 28–41. 10.1038/nrmicro3367 25417656 PMC5619696

[B18] El-Shesheny R. Feeroz M. M. Krauss S. Vogel P. Mckenzie P. Webby R. J. (2018). Replication and pathogenic potential of influenza A virus subtypes H3, H7, and H15 from free-range ducks in Bangladesh in mammals. *Emerg. Microbes Infect.* 7:70. 10.1038/s41426-018-0072-7 29691394 PMC5915612

[B19] Finkelstein D. B. Mukatira S. Mehta P. K. Obenauer J. C. Su X. Webster R. G. (2007). Persistent host markers in pandemic and H5N1 influenza viruses. *J. Virol.* 81 10292–10299. 10.1128/JVI.00921-07 17652405 PMC2045501

[B20] Govorkova E. A. Baranovich T. Seiler P. Armstrong J. Burnham A. Guan Y. (2013). Antiviral resistance among highly pathogenic influenza A (H5N1) viruses isolated worldwide in 2002-2012 shows need for continued monitoring. *Antivir. Res.* 98 297–304. 10.1016/j.antiviral.2013.02.013 23458714 PMC3648604

[B21] Graef K. M. Vreede F. T. Lau Y.-F. Mccall A. W. Carr S. M. Subbarao K. (2010). The PB2 subunit of the influenza virus RNA polymerase affects virulence by interacting with the mitochondrial antiviral signaling protein and inhibiting expression of beta interferon. *J. Virol.* 84:8433. 10.1128/JVI.00879-10 20538852 PMC2919034

[B22] Heider H. Adamczyk B. Presber H. W. Schroeder C. Feldblum R. Indulen M. K. (1981). Occurrence of amantadine- and rimantadine-resistant influenza A virus strains during the 1980 epidemic. *Acta Virol.* 25 395–400. 6120642

[B23] Hinshaw V. S. Bean W. J. Webster R. G. Sriram G. (1980). Genetic reassortment of influenza A viruses in the intestinal tract of ducks. *Virology* 102 412–419. 10.1016/0042-6822(80)90108-7 6245516

[B24] Hussain M. Galvin H. D. Haw T. Y. Nutsford A. N. Husain M. (2017). Drug resistance in influenza A virus: the epidemiology and management. *Infect. Drug Resist.* 10 121–134. 10.2147/IDR.S105473 28458567 PMC5404498

[B25] Jalily P. H. Duncan M. C. Fedida D. Wang J. Tietjen I. (2020). Put a cork in it: plugging the M2 viral ion channel to sink influenza. *Antivir. Res.* 178:104780. 10.1016/j.antiviral.2020.104780 32229237 PMC7102647

[B26] Järhult J. D. (2012). Oseltamivir (Tamiflu(^®^)) in the environment, resistance development in influenza A viruses of dabbling ducks and the risk of transmission of an oseltamivir-resistant virus to humans–a review. *Infect. Ecol. Epidemiol.* 2 10.3402/iee.v2i0.18385 22957124 PMC3426320

[B27] Joseph U. Su Y. C. F. Vijaykrishna D. Smith G. J. D. (2017). The ecology and adaptive evolution of influenza A interspecies transmission. *Influenza Other Respir. Viruses* 11 74–84. 10.1111/irv.12412 27426214 PMC5155642

[B28] Kaul K. L. Mangold K. A. Du H. Pesavento K. M. Nawrocki J. Nowak J. A. (2010). Influenza A subtyping: seasonal H1N1, H3N2, and the appearance of novel H1N1. *J. Mol. Diagn.* 12 664–669. 10.2353/jmoldx.2010.090225 20595627 PMC2928431

[B29] Kim J. H. Hatta M. Watanabe S. Neumann G. Watanabe T. Kawaoka Y. (2010). Role of host-specific amino acids in the pathogenicity of avian H5N1 influenza viruses in mice. *J. Gen. Virol.* 91 1284–1289. 10.1099/vir.0.018143-0 20016035 PMC2878586

[B30] Kim S. H. (2018). Challenge for one health: co-circulation of zoonotic H5N1 and H9N2 avian influenza viruses in Egypt. *Viruses* 10:121. 10.3390/v10030121 29522492 PMC5869514

[B31] Komadina N. Mcvernon J. Hall R. Leder K. (2014). A historical perspective of influenza A(H1N2) virus. *Emerg. Infect. Dis.* 20 6–12. 10.3201/eid2001.121848 24377419 PMC3884707

[B32] Lai S. Qin Y. Cowling B. J. Ren X. Wardrop N. A. Gilbert M. (2016). Global epidemiology of avian influenza A H5N1 virus infection in humans, 1997-2015: a systematic review of individual case data. *Lancet Infect. Dis.* 16 e108–e118. 10.1016/S1473-3099(16)00153-5 27211899 PMC4933299

[B33] Lynch J. P. III Walsh E. E. (2007). Influenza: evolving strategies in treatment and prevention. *Semin Respir. Crit. Care Med.* 28 144–158. 10.1055/s-2007-976487 17458769

[B34] Mao L. Yang Y. Qiu Y. Yang Y. (2012). Annual economic impacts of seasonal influenza on US counties: spatial heterogeneity and patterns. *Int. J. Health Geogr.* 11:16. 10.1186/1476-072X-11-16 22594494 PMC3479051

[B35] Marinova-Petkova A. Shanmuganatham K. Feeroz M. M. Jones-Engel L. Hasan M. K. Akhtar S. (2016). The continuing evolution of H5N1 and H9N2 influenza viruses in Bangladesh between 2013 and 2014. *Avian Dis.* 60 108–117. 10.1637/11136-050815-Reg 27309046 PMC5479493

[B36] McCauley J. W. Mahy B. W. (1983). Structure and function of the influenza virus genome. *Biochem. J.* 211 281–294. 10.1042/bj2110281 6191756 PMC1154358

[B37] Mehle A. Doudna J. A. (2008). An inhibitory activity in human cells restricts the function of an avian-like influenza virus polymerase. *Cell Host Microbe* 4 111–122. 10.1016/j.chom.2008.06.007 18692771 PMC2597520

[B38] Mehle A. Doudna J. A. (2009). Adaptive strategies of the influenza virus polymerase for replication in humans. *Proc. Natl. Acad. Sci. U.S.A.* 106 21312–21316. 10.1073/pnas.0911915106 19995968 PMC2789757

[B39] Moasser E. Moasser A. Zaraket H. (2019). Incidence of antiviral drug resistance markers among human influenza A viruses in the Eastern Mediterranean region, 2005-2016. *Infect. Genet. Evol.* 67 60–66. 10.1016/j.meegid.2018.10.023 30389548

[B40] Molinari N. A. Ortega-Sanchez I. R. Messonnier M. L. Thompson W. W. Wortley P. M. Weintraub E. (2007). The annual impact of seasonal influenza in the US: measuring disease burden and costs. *Vaccine* 25 5086–5096. 10.1016/j.vaccine.2007.03.046 17544181

[B41] Mostafa A. Abdelwhab E. M. Mettenleiter T. C. Pleschka S. (2018). Zoonotic potential of influenza A viruses: a comprehensive overview. *Viruses* 10:497. 10.3390/v10090497 30217093 PMC6165440

[B42] Musharrafieh R. Ma C. Wang J. (2020). Discovery of M2 channel blockers targeting the drug-resistant double mutants M2-S31N/L26I and M2-S31N/V27A from the influenza A viruses. *Eur. J. Pharm. Sci.* 141:105124. 10.1016/j.ejps.2019.105124 31669761 PMC6951800

[B43] Nelson M. I. Simonsen L. Viboud C. Miller M. A. Holmes E. C. (2009). The origin and global emergence of adamantane resistant A/H3N2 influenza viruses. *Virology* 388 270–278. 10.1016/j.virol.2009.03.026 19394063 PMC2705899

[B44] Nooruzzaman M. Mumu T. T. Hasnat A. Akter M. N. Rasel M. S. U. Rahman M. M. (2019). A new reassortant clade 2.3.2.1a H5N1 highly pathogenic avian influenza virus causing recent outbreaks in ducks, geese, chickens and turkeys in Bangladesh. *Transbound. Emerg. Dis.* 66 2120–2133. 10.1111/tbed.13264 31168925

[B45] Ormond L. Liu P. Matuszewski S. Renzette N. Bank C. Zeldovich K. (2017). The combined effect of oseltamivir and favipiravir on influenza A virus evolution. *Genome Biol. Evol.* 9 1913–1924. 10.1093/gbe/evx138 28854600 PMC5570085

[B46] Orozovic G. Orozovic K. Lennerstrand J. Olsen B. (2011). Detection of resistance mutations to antivirals oseltamivir and zanamivir in avian influenza A viruses isolated from wild birds. *PLoS One* 6:e16028. 10.1371/journal.pone.0016028 21253602 PMC3017088

[B47] Palekar R. S. Rolfes M. A. Arriola C. S. Acosta B. O. Guidos P. A. Vargas X. B. (2019). Burden of influenza-associated respiratory hospitalizations in the Americas, 2010–2015. *PLoS One* 14:e0221479. 10.1371/journal.pone.0221479 31490961 PMC6730873

[B48] Parida M. Dash P. K. Kumar J. S. Joshi G. Tandel K. Sharma S. (2016). Emergence of influenza A (H1N1)pdm09 genogroup 6B and drug resistant virus, India, January to May 2015. *Euro Surveill.* 21 6–11. 10.2807/1560-7917.ES.2016.21.5.30124 26876980

[B49] Parvin R. Begum J. A. Nooruzzaman M. Chowdhury E. H. Islam M. R. Vahlenkamp T. W. (2018). Review analysis and impact of co-circulating H5N1 and H9N2 avian influenza viruses in Bangladesh. *Epidemiol. Infect.* 146 1259–1266. 10.1017/S0950268818001292 29781424 PMC9134290

[B50] Parvin R. Heenemann K. Halami M. Y. Chowdhury E. H. Islam M. R. Vahlenkamp T. W. (2014). Full-genome analysis of avian influenza virus H9N2 from Bangladesh reveals internal gene reassortments with two distinct highly pathogenic avian influenza viruses. *Arch. Virol.* 159 1651–1661. 10.1007/s00705-014-1976-8 24420161

[B51] Paules C. I. Fauci A. S. (2019). Influenza vaccines: good, but we can do better. *J. Infect. Dis.* 219 S1–S4. 10.1093/infdis/jiy633 30715469 PMC6787547

[B52] Principi N. Camilloni B. Alunno A. Polinori I. Argentiero A. Esposito S. (2019). Drugs for influenza treatment: is there significant news? *Front. Med.* 6:109. 10.3389/fmed.2019.00109 31192211 PMC6546914

[B53] Proença-Módena J. L. Macedo I. S. Arruda E. (2007). H5N1 avian influenza virus: an overview. *Braz. J. Infect. Dis.* 11 125–133. 10.1590/S1413-86702007000100027 17625741

[B54] Rimi A. N. Hassan Z. M. Chowdhury S. Rahman M. Sultana R. Biswas K. P. (2019). A decade of avian influenza in Bangladesh: where are we now? *Trop. Med. Infect. Dis.* 4:119. 10.3390/tropicalmed4030119 31514405 PMC6789720

[B55] Sharp G. B. Kawaoka Y. Jones D. J. Bean W. J. Pryor S. P. Hinshaw V. (1997). Coinfection of wild ducks by influenza A viruses: distribution patterns and biological significance. *J. Virol.* 71 6128–6135. 10.1128/jvi.71.8.6128-6135.1997 9223507 PMC191873

[B56] Shin W. J. Seong B. L. (2019). Novel antiviral drug discovery strategies to tackle drug-resistant mutants of influenza virus strains. *Expert. Opin. Drug Discov.* 14 153–168. 10.1080/17460441.2019.1560261 30585088

[B57] Song W. Wang P. Mok B. W.-Y. Lau S.-Y. Huang X. Wu W.-L. (2014). The K526R substitution in viral protein PB2 enhances the effects of E627K on influenza virus replication. *Nat. Commun.* 5:5509. 10.1038/ncomms6509 25409547 PMC4263149

[B58] Suzuki Y. (2005). Sialobiology of influenza: molecular mechanism of host range variation of influenza viruses. *Biol. Pharm. Bull.* 28 399–408. 10.1248/bpb.28.399 15744059

[B59] Takeda M. Pekosz A. Shuck K. Pinto L. H. Lamb R. A. (2002). Influenza a virus M2 ion channel activity is essential for efficient replication in tissue culture. *J. Virol.* 76 1391–1399. 10.1128/JVI.76.3.1391-1399.2002 11773413 PMC135863

[B60] Taubenberger J. K. Kash J. C. (2010). e>Influenza virus evolution, host adaptation, and pandemic formation. *Cell Host Microbe* 7 440–451. 10.1016/j.chom.2010.05.009 20542248 PMC2892379

[B61] Thomas J. K. Noppenberger J. (2007). Avian influenza: a review. *Am. J. Health Syst. Pharm.* 64 149–165. 10.2146/ajhp060181 17215466

[B62] Tian J. Qi W. Li X. He J. Jiao P. Zhang C. (2012). A single E627K mutation in the PB2 protein of H9N2 avian influenza virus increases virulence by inducing higher glucocorticoids (GCs) level. *PLoS One* 7:e38233. 10.1371/journal.pone.0038233 22719870 PMC3374829

[B63] Toots M. Plemper R. K. (2020). Next-generation direct-acting influenza therapeutics. *Transl. Res.* 220 33–42. 10.1016/j.trsl.2020.01.005 32088166 PMC7102518

[B64] Trebbien R. Larsen L. E. Viuff B. M. (2011). Distribution of sialic acid receptors and influenza A virus of avian and swine origin in experimentally infected pigs. *Virol. J.* 8:434. 10.1186/1743-422X-8-434 21902821 PMC3177912

[B65] Turner J. C. Feeroz M. M. Hasan M. K. Akhtar S. Walker D. Seiler P. (2017). Insight into live bird markets of Bangladesh: an overview of the dynamics of transmission of H5N1 and H9N2 avian influenza viruses. *Emerg. Microbes Infect.* 6:e12. 10.1038/emi.2016.142 28270655 PMC5378921

[B66] Vanderlinden E. Naesens L. (2014). Emerging antiviral strategies to interfere with influenza virus entry. *Med. Res. Rev.* 34 301–339. 10.1002/med.21289 23801557 PMC7168512

[B67] Wang P. Song W. Mok B. W. Zheng M. Lau S. Y. Liu S. (2019). The PB2 polymerase host adaptation substitutions prime avian indonesia sub clade 2.1 H5N1 viruses for infecting humans. *Viruses* 11:292. 10.3390/v11030292 30909490 PMC6480796

[B68] Wen L. Chu H. Wong B. H. Wang D. Li C. Zhao X. (2018). Large-scale sequence analysis reveals novel human-adaptive markers in PB2 segment of seasonal influenza A viruses. *Emerg. Microbes Infect.* 7:47. 10.1038/s41426-018-0050-0 29593225 PMC5874250

[B69] WHO (2018). Summary of Neuraminidase Amino Acid Substitutions Associated With Reduced Inhibition by Neuraminidase Inhibitors. Available online at: https://www.who.int/influenza/gisrs_laboratory/antiviral_susceptibility/NAI_Reduced_Susceptibility_Marker_Table_WHO.pdf?ua=1 (accessed 30 June, 2021).

[B70] Wilcox B. R. Knutsen G. A. Berdeen J. Goekjian V. Poulson R. Goyal S. (2011). Influenza-A viruses in ducks in northwestern minnesota: fine scale spatial and temporal variation in prevalence and subtype diversity. *PLoS One* 6:e24010. 10.1371/journal.pone.0024010 21931636 PMC3172203

[B71] Wilson J. C. von Itzstein M. (2003). Recent strategies in the search for new anti-influenza therapies. *Curr. Drug Targets* 4 389–408. 10.2174/1389450033491019 12816348

[B72] Yamada S. Hatta M. Staker B. L. Watanabe S. Imai M. Shinya K. (2010). Biological and structural characterization of a host-adapting amino acid in influenza virus. *PLoS Pathog.* 6:e1001034. 10.1371/journal.ppat.1001034 20700447 PMC2916879

[B73] Yin H. Jiang N. Shi W. Chi X. Liu S. Chen J.-L. (2021). Development and effects of influenza antiviral drugs. *Molecules* 26:810. 10.3390/molecules26040810 33557246 PMC7913928

[B74] Yoo S. J. Kwon T. Lyoo Y. S. (2018). Challenges of influenza A viruses in humans and animals and current animal vaccines as an effective control measure. *Clin. Exp. Vaccine Res.* 7 1–15. 10.7774/cevr.2018.7.1.1 29399575 PMC5795040

[B75] Zhang Y. Aevermann B. D. Anderson T. K. Burke D. F. Dauphin G. Gu Z. (2016). Influenza research database: an integrated bioinformatics resource for influenza virus research. *Nucleic Acids Res.* 45 D466–D474. 10.1093/nar/gkw857 27679478 PMC5210613

[B76] Zhou H. Zhang A. Chen H. Jin M. (2011). Emergence of novel reassortant H3N2 influenza viruses among ducks in China. *Arch. Virol.* 156 1045–1048. 10.1007/s00705-011-0940-0 21318308

[B77] Zürcher T. Yates P. J. Daly J. Sahasrabudhe A. Walters M. Dash L. (2006). Mutations conferring zanamivir resistance in human influenza virus N2 neuraminidases compromise virus fitness and are not stably maintained in vitro. *J. Antimicrob. Chemother.* 58 723–732. 10.1093/jac/dkl321 16891631

